# Thermal asymmetry in the Moon’s mantle inferred from monthly tidal response

**DOI:** 10.1038/s41586-025-08949-5

**Published:** 2025-05-14

**Authors:** R. S. Park, A. Berne, A. S. Konopliv, J. T. Keane, I. Matsuyama, F. Nimmo, M. Rovira-Navarro, M. P. Panning, M. Simons, D. J. Stevenson, R. C. Weber

**Affiliations:** 1 Jet Propulsion Laboratory, California Institute of Technology, Pasadena, CA USA; 2 Seismological Laboratory, California Institute of Technology, Pasadena, CA USA; 3 Lunar and Planetary Laboratory, University of Arizona, Tucson, AZ USA; 4 Department of Earth and Planetary Sciences, University of California, Santa Cruz, CA USA; 5 Delft University of Technology, Delft, the Netherlands; 6 Division of Geological and Planetary Science, California Institute of Technology, Pasadena, CA USA; 7 NASA Marshall Space Flight Center, Huntsville, AL USA

**Keywords:** Planetary science, Geodynamics, Rings and moons

## Abstract

The Moon undergoes periodic tidal forcing due to its eccentric and oblique orbit around the Earth^1^. The response to this tidal interaction drives temporal changes in the lunar gravity field and is sensitive to the satellite’s internal structure^2–4^. We use data from the NASA GRAIL spacecraft^5–9^ to recover the time-varying lunar gravity field, including a degree-3 gravitational tidal Love number, *k*_3_. Here, we report our estimated value of *k*_3_ = 0.0163 ± 0.0007, which is about 72% higher than that expected for a spherically symmetric moon^10^. Such a large *k*_3_ can be explained if the elastic shear modulus of the mantle varies by about 2–3% between the nearside and farside^4^, providing an observational demonstration of lateral heterogeneities in the deep lunar interior. This asymmetric structure suggests preservation of a predominantly thermal anomaly of roughly 100–200 K in the nearside mantle that formed surface mare regions 3–4 billion years ago^11^ and could influence the spatial distribution of deep moonquakes^12^.

## Abstract (web summary)

Data from the NASA GRAIL spacecraft recover the lunar gravity field suggesting preservation of a predominantly thermal anomaly in the nearside mantle, which could influence the spatial distribution of deep moonquakes.

## Main

The Moon shows well-known nearside–farside differences, reflected in the offset between its centre of mass and centre of figure (COM–COF), as well as asymmetries in topography, crustal thickness, surface concentration of radiogenic elements and geology^13^. Various hypotheses have been proposed to explain this asymmetry, although its origin remains widely debated. Some studies suggest that the Moon’s nearside–farside asymmetries are linked to variations in its deep internal structure, including the distribution of radiogenic heat-producing elements, which could sustain long-lived temperature differences between the nearside and farside^11,14,15^. These models can explain the concentration of volcanism on the Moon’s nearside and provide constraints on the poorly understood bulk concentrations of lunar radiogenic elements^16^. However, so far, no observational evidence for such temperature differences or variations in deep internal structure has been unambiguously detected. In this study, we aim to determine the magnitude of these differences at depth by analysing the Moon’s gravitational response to its periodic tidal interactions with Earth.

The gravity field of the Moon is typically expressed in terms of spherical harmonic coefficients of degree *l* and order *m* (ref. ^17^). The spatial resolution of the gravity field is inversely proportional to *l*, with the full wavelength usually defined as roughly 2π*R*/*l*, where lunar radius *R* = 1,738 km. Temporal changes in the lunar gravity field can be quantified using gravitational tidal Love numbers, *k*_*lm*_, which represent the ratio of the induced potential from the deformation of the Moon to the imposed gravitational potential from Earth at a given degree and order^18^. Thus, *k*_*lm*_ scales the lunar gravity field as the relative positions of the Moon and Earth vary over the course of a month.

**Extended Data Fig. 1:**
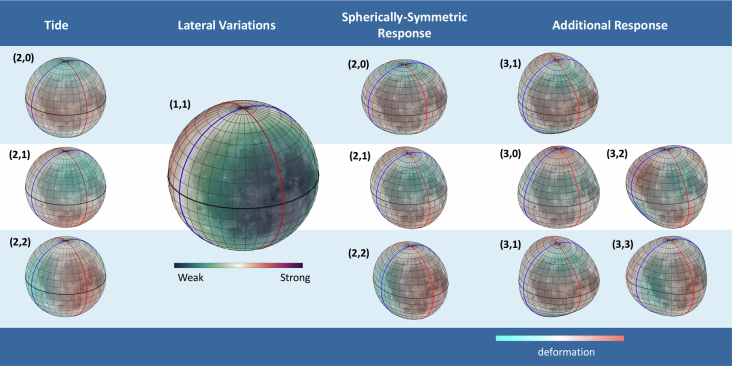
Conceptual relationship between lunar 3D structure and response to tidal forcing. The left column shows the spatial pattern of gravitational potentials associated with eccentricity tides at degree-2, order-0,2 (i.e., (2,0) and (2,2)) and obliquity tides degree-2, order-1 (2,1) acting on the Moon. The second column shows the spatial pattern of a degree-1, order-1 (i.e., (1,1) or nearside farside) lateral heterogeneity in shear modulus imposed onto the lunar interior. The third column shows response at (2,0), (2,1), and (2,2) expected for a spherically symmetric interior (i.e., also the main components of the response for a laterally heterogeneous interior). The fourth and fifth columns show additional modes of deformation at degree-3 (i.e., (3,0), (3,1), (3,2), and (3,3)) expected for the laterally heterogeneous interior shown in the second column. Rows in the third, fourth, and fifth columns correspond to response associated with forcing in the same row in column 1. For example, (2,1) forcing stimulates response at (3,0) and (3,2), (2,0) forcing stimulates response at (3,1), and (2,2) forcing stimulates response at (3,1) and (3,3).

For spherically symmetric bodies, forcing at a given degree and order induces deformation only at the same degree and order. However, if the Moon is laterally heterogeneous, then forcing at a given degree and order can drive deformation at other degrees and orders^19^. A laterally heterogeneous moon subject to tidal forcing at *l* = 2 will therefore show deformation at all degrees (*l* ≥ *2*), as well as anomalous degree-3 Love numbers (*k*_3*m*_) (Extended Data Fig. 1). Moreover, unlike with static gravity—which is most sensitive to structures in the uppermost crust—time-varying long-wavelength gravity is strongly sensitive to deep-seated asymmetries in the lunar mantle^20^. This sensitivity to deep lateral heterogeneity makes the analysis of gravitational tidal Love numbers a powerful tool for probing the structure of the lunar interior^4,15^.

## Measuring the Moon’s time-varying gravity

To determine the gravitational tidal Love numbers for the Moon, we recover its time-varying gravity field using satellite-to-satellite and Deep Space Network (DSN) radiometric tracking data acquired by NASA’s Gravity Recovery and Interior Laboratory (GRAIL) mission^9^. The GRAIL mission consisted of two orbiters, Ebb and Flow, and had two science phases: the Primary Mission (PM) and the Extended Mission (XM). We analyse both PM and XM radiometric data using the Jet Propulsion Laboratory’s (JPL’s) latest-available Development Ephemeris 440 (DE440) for Moon’s orbit and orientation^1^ as well as the previously released DE430 (ref. ^21^). Estimated global parameters include coefficients of an *l* = 1,800 static gravity field (that is, roughly 3.24 million parameters and full wavelength resolution of 6 km) using DE430 and a subset *l* = 1,200 gravity field for the DE440 solution (Methods). In the latter case, the *l* = 1,201–1,800 gravity coefficients are fixed to values from the DE430 solution to compute *k*_2*m*_ and *k*_3*m*_ at the monthly period. Our full *l* = 1,800 static gravity field is called GL1800F and is accurate to roughly *l* = 700–900 (with an average accuracy of *l* = 850), depending primarily on the latitude, with improved correlations of the covariance between short- and long-wavelength (for example, *l* = 2 and *l* = 3) gravitational signals, including tides (Methods).

Several studies have provided gravity field and Love number estimates based on GRAIL data^5–8,22–24^, generally only solving for *l* = 2 and *l* = 3 Love numbers^5,7^. Degree-3 Love numbers at different orders are assumed to be equal in these past studies (that is, *k*_3_ = *k*_30_ = *k*_31_ = *k*_32_ = *k*_33_) simplifying this parameter to just *k*_3_. In ref. ^5^, time-varying lunar gravity fields are derived by co-estimating Love numbers alongside a *l* = 100 static gravity field using the GRAIL PM data, yielding *k*_3_ = 0.0089 ± 0.0021. In ref. ^7^, estimates of *l* = 3 Love numbers also incorporated PM-only gravity data and reported *k*_30_ = 0.00734 ± 0.0015. Tidal *k*_3_ estimates based on higher resolution static gravity fields derived from both PM and XM data were not reported previously from the GRAIL project, primarily because the Moon was assumed to be spherically symmetric^10^ and the high *k*_3_ values computed using both datasets were considered unrealistic. Put simply, *k*_3_ was not expected to have substantial scientific value, so methodological factors that may have biased this parameter were not seriously examined.

**Fig. 1:**
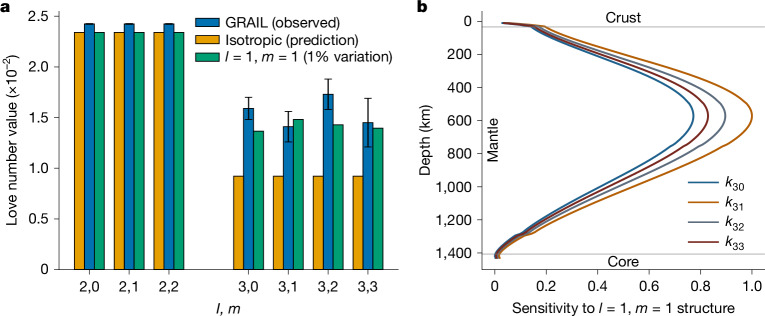
Sensitivity of lunar gravitational tidal Love numbers to laterally heterogeneous structure. **a**, Bar chart showing *k*_2*m*_ and *k*_3*m*_ values expected for an isotropic moon (orange), observed Love number values with 15× formal 1-*σ* uncertainties error bars (blue) and values predicted for a lunar interior with an imposed 1% nearside–farside (*l* = 1, *m* = 1) variation in mantle shear modulus (green). Love numbers for the isotropic case represent values predicted for the 1D lunar interior derived from seismic travel-time data in ref. ^27^. **b**, Normalized sensitivity of *k*_30_ (blue), *k*_31_ (orange), *k*_32_ (grey) and *k*_33_ (red) Love numbers to (*l* = 1, *m* = 1) perturbations (that is, a nearside–farside pattern) in shear modulus placed at depths ranging from the surface (0 km) to the core-mantle boundary (1,407 km) for reference lunar interiors^27^ subject to *l* = 2 forcing (for example, eccentricity tides expected for the lunar orbit). Labels refer to vertical regions spanning the crust (0–34 km), the mantle (34–1,407 km) and the core (1,407–1737 km).

With GL1800F, we recover *k*_2*m*_ and *k*_3*m*_ for two separate cases, which are shown in Table 1. In the first case, *l* = 3 Love numbers are assumed not to depend on *m* and we recover *k*_3_ = *k*_3*m*_ = 0.0163 ± 0.0007. In the second case, *k*_3*m*_ are estimated independently yielding *k*_30_ = 0.0159 ± 0.0011, *k*_31_ = 0.0141 ± 0.0015, *k*_32_ = 0.0173 ± 0.0015 and *k*_33_ = 0.0145 ± 0.0024 (Table 1 and Fig. 1a). Our recovered *k*_3_ and *k*_3*m*_ are substantially larger than previously reported *k*_3_ values^5,7^ due primarily to our inclusion of low-altitude XM data. The XM data substantially improves the full harmonic range of gravity information, which results in reduction of correlations between the gravity field coefficients and *k*_3*m*_ (Methods). For example, we find that *k*_3_ computed with gravity fields that only use the PM data (that is, the full GL0660B model but with an estimated subset gravity field to *l* = 150) is *k*_3_ = 0.0098 ± 0.0021, which is consistent with previously reported values of *k*_3_ = 0.0089 ± 0.0021 (ref. ^5^) and *k*_30_ = 0.00734 ± 0.0015 (ref. ^7^) with a degree and order 660 gravity field. Evaluating static gravity fields up to a higher degree (for example, *l* > 420) using PM data, or empirically tightening constraints on non-gravitational accelerations for inversions (for example, fig. 9 of ref. ^5^), also increases *k*_3_ relative to values reported in previous studies. This indicates that improving the correlation between *k*_3*m*_ and *l* > 150 gravity harmonics by adding the XM data has a key role in the ability to recover accurate estimates of *k*_3*m*_.

**Table 1:** Recovered gravitational tidal Love numbers, *k*_2*m*_ and *k*_3*m*_, from the GL1800F solution

	Parameter	Value	15× formal 1-*σ*	Notes
Case 1	*k*_20_	0.024223	0.000037	Nominal GL1800F solution. The expected values of *k*_2_ and *k*_3_ for a spherically symmetric moon are 0.0234 and 0.00945, respectively^10,27^.
	*k*_21_	0.024223	0.000037	
	*k*_22_	0.024223	0.000037	
	*k*_3_	0.0163	0.0007	
Case 2	*k*_20_	0.024237	0.000037	Estimated *l* = 150 gravity field with *k*_3*m*_ with GL1800F as the background static gravity field.
	*k*_21_	0.024236	0.000037	
	*k*_22_	0.024236	0.000037	
	*k*_30_	0.0159	0.0011	
	*k*_31_	0.0141	0.0015	
	*k*_32_	0.0173	0.0015	
	*k*_33_	0.0145	0.0024	

Case 1 shows the *k*_2*m*_ and *k*_3_ values from the nominal GL1800F solution. The recovered *k*_3_ = 0.0163 ± 0.0007 is roughly 72% larger than the value expected for a spherically symmetric moon (Fig. 1a). Case 2 shows individual *k*_2*m*_ and *k*_3*m*_ when values at each order are estimated independently with a degree-150 gravity field using GL1800F as the background static gravity field. The magnitudes of the recovered order-dependent *k*_3*m*_ are comparable to the recovered *k*_3_ value in case 1.

## Modelling the Moon’s internal structure

**Extended Data Fig. 2:**
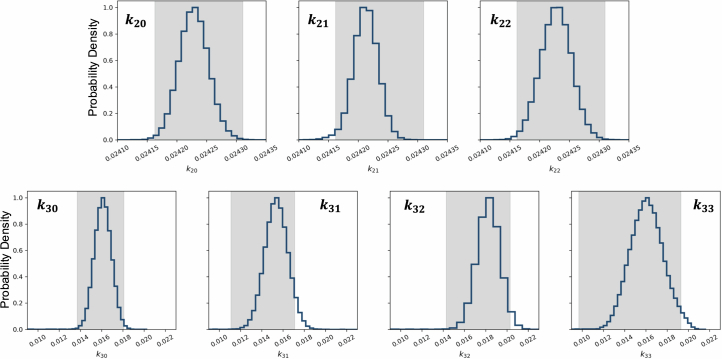
Distributions of modeled Love numbers *k*_2*m*_ and *k*_3*m*_ corresponding to the ensemble of accepted candidate models. Grey boxes represent observational constraints, where the box width is 15 times the formal uncertainty for each value as presented in Table 1.

**Extended Data Fig. 3:**
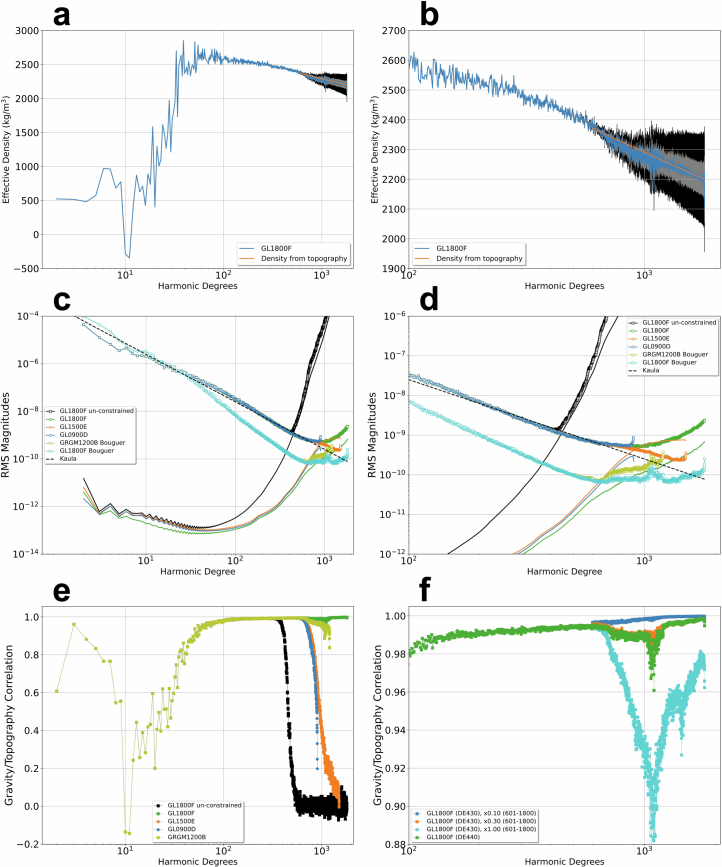
Effective density, gravity spectrums, and global correlations of gravity fields with gravity inferred from topography as a function of harmonic degree. **a**, The effective density of GL1800F (blue) is computed using the admittance of gravity and gravity-from-topography spherical harmonics. The orange line shows the power law fit of the effective density from degrees 200 to 600 and then extrapolated to a density of 2,200 kg/m^3^ at *l* = 1800. The uncertainty for the effective density was computed from 10,000 clones for *l* = 2–1200, and extrapolated for *l* = 1201–1800, which is shown in gray (1-*σ*) and black (3-*σ*). **b**, Zoomed in version of **a**. **c**, Full gravity spectrum of both constrained and unconstrained solutions are shown and compared to previously released lunar gravity fields. The circles represent the RMS and solid lines represent the corresponding uncertainty. The GL1800F (green) shows a significant improvement over GL0900D. Note that GL0900D shows a smaller uncertainty for *l* = 2 compared to GL1500E and GL1800F because *k*_2m_ and *k*_3m_ were not estimated in GL0900D. The Bouguer spectrum of GL1800F (cyan) shows that the solution is on average accurate to approximately *l* = 850. **d**, Zoomed in version of **c**. **e**, Correlations of GL1800F, GL1500E, GL0900D, and GRGM1200B are shown. Correlation of the un-constrained GL1800F solution is also shown for comparison. Similar to the gravity spectrum shown in Extended Data Fig. 2c, GL1800F shows a significant improvement over GL0900D. **f**, Correlation of GL1800F is compared with other *l* = 1800 solutions using DE430 and constraint of the higher degrees of the lunar gravity field to within 10% (blue), 30% (orange), and 100% (cyan). We use the 30% constraint as the baseline solution for GL1800F.

**Extended Data Fig. 4:**
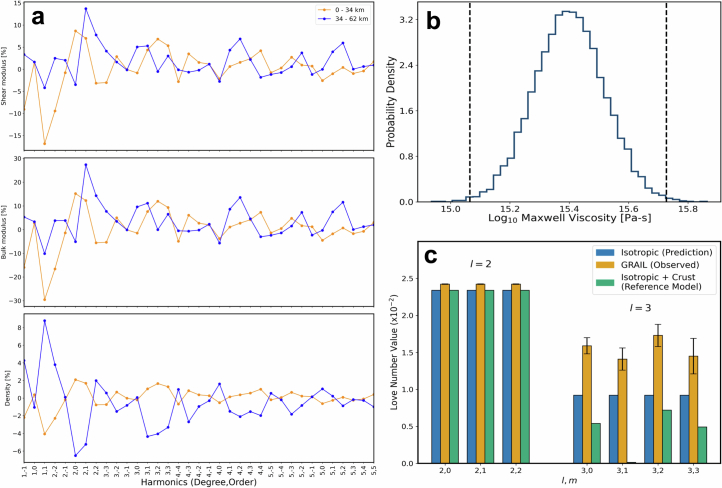
Description of reference model and low velocity zone viscosity. **a**, Lateral variations in crustal structure assumed for reference models. Line plots with scatter points show shear modulus (top), bulk modulus (middle), and density (bottom) perturbations (parameterized as spherical harmonic coefficients up to degree-5, normalized as a percentage of the mean value (see Eq. 6)) for two vertical regions of the lunar interior spanning 0–34 km (orange line) and 34–62 km (blue line). Coefficient values derived linearly mapping lateral variations in crustal properties from ref. ^25^ to variations in bulk/shear moduli and density (see Eqn. 75 of ref. ^20^). Note that density variations reflect both observed variations in the density of the crust^25^ and effective density variations due to lateral variability in the depth of the Moho whereas bulk/shear moduli variations only reflect variations in Moho depth. **b**, Inferred Maxwell viscosity for 1,257–1,407 km depth. Histogram shows Maxwell viscosity values corresponding to the inverted reduction in the degree-0 shear modulus value of the region spanning 1,257–1,407 km depth (Extended Data Table 2 and Eq. 8). Dashed lines show 0.003, and 0.997 quantiles (i.e., 3-*σ* confidence bounds). **c**, Similar to Fig. 1b except including models with crustal thickness and density variations. Note that the green bar above the *l* = 3, *m* = 1 harmonic is very close to zero.

Our recovered *k*_3_ is roughly 72% larger than the value expected for a spherically symmetric interior^10^ (Fig. 1a and Methods), suggesting substantial lateral heterogeneity within the Moon (Fig. 1b). To constrain the nature of this asymmetry, we perform a Markov chain Monte Carlo (MCMC) inversion to predict *k*_2*m*_ and *k*_3*m*_ (Extended Data Fig. 2 and Methods) using the observational constraints shown in case 2 of Table 1. The parameter set we explore includes shear modulus perturbations to a one-dimensional (1D) reference model derived from seismic travel-time data (Extended Data Table 1). The reference models also incorporate lateral crustal thickness and density variations derived from lunar static gravity and topography data^25^ (Extended Data Figs. 3 and 4a,c) but do not include lateral variations in shear modulus a priori for any layer. We use spherical harmonics up to *l* = 3 to parameterize perturbations for two internal layers: the crust (0–34 km depth) and the mantle (34–1,407 km depth). Lateral heterogeneity in the core minimally affects lunar time-variable gravity fields (Fig. 1b); therefore, models assume a laterally homogeneous elastic structure below 1,407 km depth. For the inversion, we use LOV3D (ref. ^3^), a semi-analytical spectral method to forward compute gravitational tidal Love numbers from candidate interior structures (Methods).

We find that positive (*l* = 1, *m* = 1) shear modulus structure, which corresponds to a nearside–farside pattern, increases all *k*_3*m*_ Love numbers for lunar interiors subject to monthly tidal forcing at *l* = 2 (green bars in Fig. 1a). For example, the combination of (*l* = 2, *m* = 2) and (*l* = 2, *m* = 0) harmonics in the Earth–Moon eccentricity tide interacts with lower (or higher) shear modulus values on the lunar nearside or farside to broadly increase (or decrease) outward radial deformation in these regions (Extended Data Fig. 1). The resulting mass displacement yields (*l* = 3, *m* = 1) and (*l* = 3, *m* = 3) gravity signatures that enhance the existing response to forcing at these harmonics, increasing the *k*_31_ and *k*_33_ Love numbers. Similarly, interaction between the (*l* = 2, *m* = 1) Earth–Moon obliquity tide and nearside–farside structure produces (*l* = 3, *m* = 0) and (*l* = 3, *m* = 2) gravity signatures that increase the *k*_30_ and *k*_32_ Love numbers (Methods). Note that the eccentricity and obliquity components of the driving tidal potential and their associated coupling to degree-3 harmonics are comparable in magnitude, resulting in similar values for *k*_3*m*_ across all values of *m*.

The depth of lunar asymmetries modulates their impact on Love number values. For example, *k*_3*m*_ show peak sensitivity to nearside–farside variations in shear modulus at roughly 600 km depth and are largely insensitive to structure close to the surface (Fig. 1b). Inversions consequently do not distinguish nearside–farside structure in the crust (less than 34 km depth) (Extended Data Table 2). Variations in crustal layer thickness are also insufficient to explain *k*_3*m*_, as the roughly 50% variation in nearside–farside Moho depth alters *l* = 3 Love numbers by only about 30% (that is, roughly 0.003) relative to values predicted for a laterally isotropic Moon. Moreover, crustal thinning below mare regions tends to increase the effective shear modulus of the nearside hemisphere and decrease degree-3 Love numbers, opposite to the observed trend of *k*_3*m*_ values in Table 1 (Extended Data Fig. 4c).

**Fig. 2:**
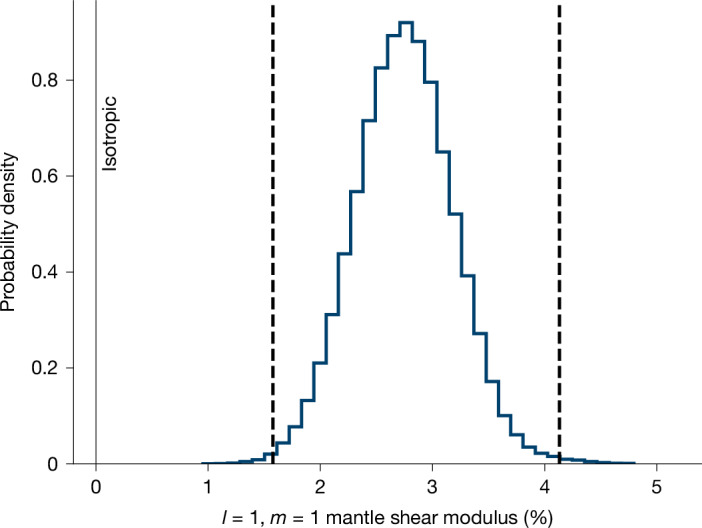
Recovered nearside–farside structure in the lunar mantle using independently recovered *k*_3*m*_. Histogram showing inverted coefficient value that describes internal (*l* = 1, *m* = 1) variations in shear modulus (in percent relative to the bulk value) for the lunar mantle. Dashed lines show 0.3 and 99.7% quantiles (that is, 3-*σ* confidence bounds). Thin vertical grey line denotes value expected for an isotropic mantle. The preferred value and 3-*σ* bounds for (*l* = 1, *m* = 1) mantle shear modulus structure is 2.74 ± 1.3%. A full list of derived harmonic coefficients describing 3D structure are shown in Extended Data Table 2.

Our inversions predict a 2–3% mean difference in shear modulus between nearside and farside hemispheres for the entire lunar mantle, with more than 99.7% confidence (Fig. 2). When we further subdivide the mantle into distinct regions for inversions, we find a slight preference for (*l* = 1, *m* = 1) structure localized to roughly the upper 800 km of the interior. This is due to the relatively higher sensitivity of *k*_3*m*_ to the *l* = 1 structure in this region (Fig. 1b). Nonetheless, the magnitude of heterogeneities derived for different mantle regions can trade off with one another to produce an overall 2–3% variation in (*l* = 1, *m* = 1) mantle shear modulus. This non-uniqueness prevents statistically significant constraints on the extent to which asymmetries localize within these layers.

In addition to affecting *k*_3*m*_, shear modulus structure is expected to drive variation in *l* = 2 Love numbers. The minimal observed differences between *k*_2*m*_ values across spherical harmonic order *m* correspondingly indicate a lack of detectable *l* = 2 variation in internal shear modulus (Fig. 1a and fig. 4 of ref. ^3^). The coupled response at degree-2 due to inferred (*l* = 1, *m* = 1) shear modulus variation (Fig. 2) also affects *k*_2*m*_ at the 10^−6^–10^−7^ level: roughly an order of magnitude smaller than observational uncertainty for these parameters (Table 1). However, the mean value of *k*_2*m*_ is roughly 5% higher than that expected for lunar interiors derived from seismic travel-time data^10,26,27^ (Fig. 1a). This increase in monthly *k*_2*m*_ can be explained by a roughly global 97% reduction in effective shear modulus at tidal timescales between depths of 1,257–1,407 km. Consistent with results from several previous studies^28,29^, this low effective shear modulus value corresponds with a local Maxwell viscosity of roughly 10^15^–10^16^ Pa s and can be explained by the presence of globally distributed partial melt in the lower mantle (Extended Data Table 2 and Extended Data Fig. 4b).

## Thermal asymmetry in the lunar mantle

**Fig. 3:**
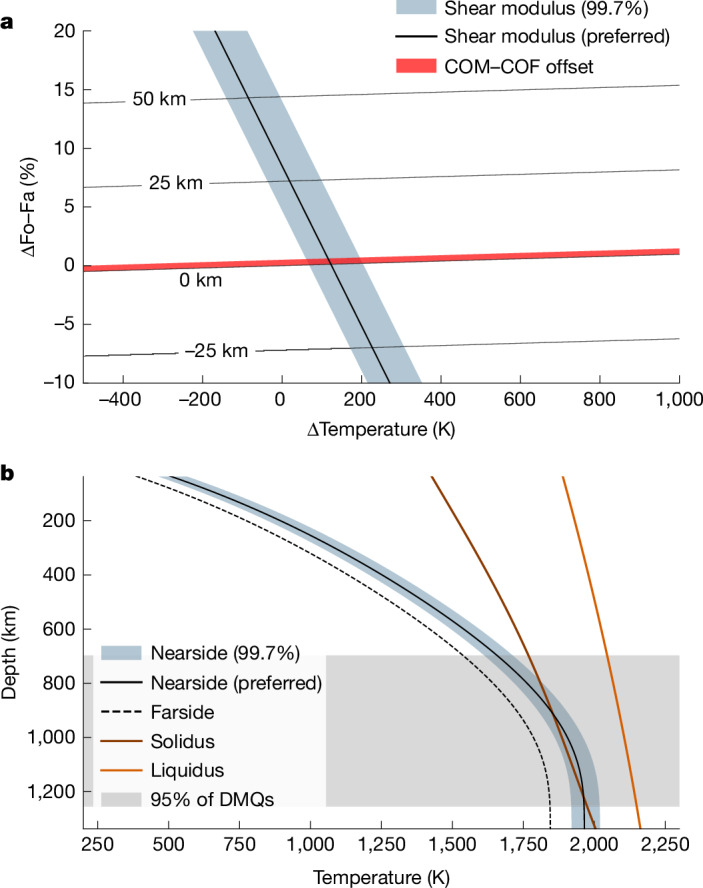
Impact of asymmetric temperature or composition on the lunar mantle. **a**, Lunar COM–COF offset and shear modulus change as a function of temperature change (assuming a volume expansion coefficient *β* = 3 × 10^−5^ K^−1^ and Δ*μ*/Δ*T* = −1.35 × 10^−2^ GPa K^−1^, where *T* is temperature and *μ* is shear modulus^33,34^) and iron content (that is, percentage changes in the mol fraction of iron-endmember olivine (fayalite (Fa)) relative to forsterite (Fo), or ΔFo–Fa, assuming Δ*ρ*/ΔFo–Fa = 9.7 kg per % per m^3^, where *ρ* is density and Δ*μ*/ΔFo–Fa = −0.3 GPa per %; ref. ^32^) in the nearside mantle. The solid red region and thin black lines respectively denote the observed COM–COF offset^31^ and contours for computed COM–COF offset values. The blue shaded area and thick solid black line, respectively, denote 99.7% confidence bounds and preferred values for the nearside–farside shear modulus differences inferred from gravity data in this work. We infer a temperature anomaly of roughly 100–200 K between the lunar near and farside hemispheres by identifying overlapping portions of the ΔFo–Fa−Δ*T* parameter space that satisfy both the shear modulus difference (within 99.7% confidence bounds) and the COM–COF offset. **b**, Internal temperature structure for the present-day lunar nearside based on predicted shear modulus change (99.7% confidence bound and preferred model are blue shaded region and solid black line, respectively). The nearside profile is computed by uniformly increasing the temperature of a reference farside conductive model for the Moon (black dashed line, extracted from fig. 5 of ref. ^11^) by the inferred 100–200 K anomaly (that is, assuming zero lateral variation in mantle composition). The lunar mantle solidus and liquidus are shown as brown and orange lines, respectively. Because the predicted nearside model exceeds the solidus, we expect present-day melt production in the lunar mantle. The grey shaded region denotes the location of 95% of observed DMQs^12^.

**Extended Data Fig. 5:**
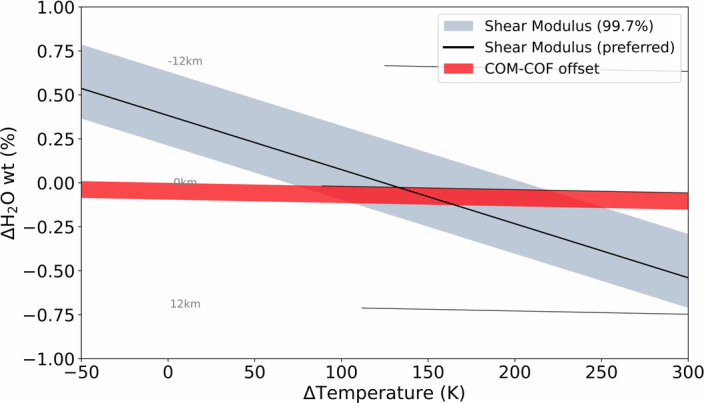
Impact of water content on mantle shear modulus. Similar to Fig. 3a except considering the impact of water content (as a weight percentage) on the shear modulus and density of lunar mantle olivine and assuming *β* = 3 × 10^−5^ K^−1^, *Δμ*/*ΔT* = −1.35 × 10^−2^ GPa/K, *Δρ*/*Δ*H_2_O = −50.56 kg/%-m^3^, and *Δμ*/*Δ*H_2_O = −6.63 GPa/% in the nearside mantle^79^. While our analysis suggests a limited water mass fraction difference of approximately 0.1% between the nearside and farside, superimposed compositional variations, such as iron and ilmenite content^2^, could influence the overall density structure and may reconcile a water-enriched nearside mantle with the small observed COM-COF offset.

Our modelling suggests that the Moon’s large *k*_3*m*_ is the result of a substantial 2–3% difference between the shear modulus of the nearside and farside mantle. What could produce this difference? At a specified pressure, the shear modulus of rock depends on its composition and temperature. However, large changes in the mantle composition probably cannot explain the derived asymmetries due to their associated impact on internal density. For example, decreasing the shear modulus by the required 2–3% solely through changes in iron content would necessitate a greater than 5% enrichment of dense iron-endmember olivine (that is, fayalite, Fa) and a corresponding increase in the density of the nearside mantle by more than 50 kg m^−^^3^ relative to the density of the farside mantle. Such a scenario would produce a COM–COF offset of the Moon that is at least 15 times larger than the observed value (Fig. 3a). Similarly, invoking a nearside enrichment in water content to explain the observed shear modulus variation would induce a COM–COF offset 5–10 times larger than the observed value^30^ (Extended Data Fig. 5). By contrast, a temperature difference of roughly 100–200 K between the two hemispheres can produce the required variation in shear modulus with a sufficiently small change in mantle density and in the COM–COF offset^31–34^. Thus, we favour a predominantly thermal explanation for these derived asymmetries.

**Fig. 4:**
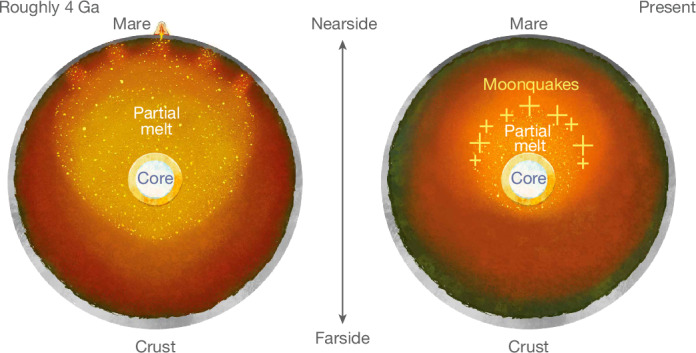
Conceptual model for the evolution of the lunar interior. Partial melt associated with the inferred nearside thermal anomaly erupts onto the surface to form mare regions roughly 4 Ga (left). As the interior cools, the partial melting associated with the inferred nearside thermal anomaly descends until localizing to depths of 800–1,200 km in the present-day (right). The colour scale denotes mantle temperature (decreasing from light yellow to dark orange to dark green). Yellow crosses denote moonquakes that localize within or slightly above partially melted regions of the present-day lunar mantle. For a similar conceptual model that also considers compositional variations in water vapour and ilmenite in the lunar interior, see fig. 4 of ref. ^2^.

It is important to note that our result indicates a present-day thermal anomaly between the nearside and farside of the lunar mantle. This hemispheric thermal dichotomy may be sustained by the high abundance of radiogenic heat sources observed in the nearside crust (or possibly at greater depths), such as thorium and titanium, which constitute a negligible mass fraction of lunar material^14,35^. Models of lunar evolution that invoke radiogenic heating as a driver for nearside–farside differences in temperature predict a partially molten mantle 3–4 billion years ago (Ga) (refs. ^11,15^). On the basis of our results, present-day magma production may still occur at 800–1,250 km depth in the nearside (Figs. 3b and 4) and further reduce the effective rigidity of this region relative to that of the deep farside interior^36^.

A persistent thermal anomaly in the lunar mantle may also influence the evolution of the overlying crust. For example, the upward migration of magma in the mantle is expected to deflect the Moho and drive crustal thinning^37^. A small fraction of this magma should also erupt on the surface to form mare regions^11^. Comparing our result with independent thermochemical evolution models^11^, we speculate that surface volcanic activity would have peaked 3–4 Ga and diminished over time as the interior cools and the depth of partial melting increases (Figs. 3b and 4). This gradual cooling of the lunar interior is consistent with the possible recent discovery of young (roughly 120 million years ago) volcanic beads from Chang’e-5 samples^38^ (for alternative hypotheses on the formation of lunar beads, see refs. ^39,40^). The formation of polygonal fractures surrounding mare regions may also accommodate the long-term thermal contraction of the nearside hemisphere (fig. 4 in ref. ^41^).

Our inferred location of partial melt (Figs. 3b and 4) coincides with the lower bound of the radial extent of deep moonquakes (DMQs)^12,20^. As previously suggested by ref. ^2^, this correlation indicates that small amounts of partial melt (less than 5% by mass) may promote brittle failure by increasing the prevalence of stress concentrations in tidally deforming regions of the lunar interior^2,12,42,43^. Small amounts of water (less than 0.1% by mass, Extended Data Fig. 5) may also reduce the freezing point of rock and further encourage seismicity in the lower mantle^2,44^. Alternatively, the enhanced ductility of very warm mantle rock may reduce its susceptibility to brittle failure and arrest DMQs at depths of roughly 1,000–1,250 km (Fig. 3b). A link between DMQs and partial melt could be tested by measuring induced components of the Moon’s magnetic field that form through interactions between the solar wind and deep magma^14^. Moreover, a mantle-wide thermal asymmetry may indicate lateral variations DMQ depth or frequency. Although few farside DMQs are observed in Apollo data^45^, it is unclear whether this is due to a difference in seismicity or due to attenuation at depth. This ambiguity may be resolved with the upcoming deployment of seismometers on the Moon with the Farside Seismic Suite planned for 2026 (ref. ^46^), the Lunar Environment Monitoring Station planned for Artemis III (ref. ^47^) and the proposed Lunar Geophysical Network mission^48^.

## Tidal tomography and future measurements

Although so-called ‘tidal tomography’ has been used to probe the deep structure of the Earth^49^, our result demonstrates an example of extraterrestrial tidal tomography. The tidal signatures of planetary bodies are generally extremely small, making them challenging to detect. However, continued advancements in measurement techniques^9,50–52^ will allow the recovery of these faint signals at a level meaningful for tidal tomography, thus providing a new way to probe the deep interior. In the future, these techniques can be applied to other planetary objects showing pronounced low-order surface variations, such as Mars^53^, Enceladus^54^ and Ganymede^55^. Because tidal tomography does not require a landed spacecraft, unlike seismology, it should be an important component of future missions that include an orbiter around the target body.

## Methods

### Analysis of GRAIL data

We analysed the PM and XM data from the GRAIL mission. The PM phase started on 1 March 2012 and ended on 29 May 2012 (that is, 89 days) with the average altitude of roughly 55 km (ref. ^5^). The XM phase started on 30 August 2012 and ended on 14 December 2012 (that is, 106 days) with the average altitude of roughly 23 km (ref. ^6^). The lower XM altitude was a key to improving the accuracy and resolution of the Moon’s static gravity field that resulted in the recovery of gravitational tidal Love numbers.

In our analysis, both PM and XM phases were divided into arcs of 2–3 days for data processing on basis of the spacecraft desaturation manoeuvre times for attitude control, so there is no thrusting during each data arc to minimize the non-gravitational errors on gravity field and Love number estimates. GRAIL’s primary dataset is the high-precision Ka-band range rate (KBRR) (32 GHz) and measurements were acquired through the onboard Lunar Gravity Ranging System^56^. The PM phase had a total of 39 arcs, and used 5-s sampled KBRR inter-satellite data and several DSN passes of two-way S-band data. The XM phase had a total of 58 arcs and used 2-s sampled KBRR data and several passes of the DSN two-way S-band data. Note that the KBRR is primarily used for determining the gravity field and in-plane spacecraft motion, whereas the DSN two-way S-band data are mainly used for determining the inertial orbit plane of the spacecraft, enabling absolute orbit positioning for GRAIL orbiters.

The separation distance between GRAIL orbiters varied linearly from 80 to 220 km near 1 April and then drifted back linearly to 80 km. This variation was designed to minimize error increases in the harmonic degrees that are in resonance with the separation distance^57^. The separation distance during XM was much smaller than the PM, ranging from 30 to 75 km, with an average of 60 km, to avoid potential multipath reflections.

### Recovery of lunar gravity field GL1800F

The gravitational field potential, $U(r,\lambda ,\phi )$, associated with the Moon is expressed as a spherical harmonic expansion^17,58^:

1 $$U(r,\lambda ,\phi )=\frac{{\rm{GM}}}{r}\mathop{\sum }\limits_{l=0}^{\infty }\mathop{\sum }\limits_{m=0}^{l}{\left(\frac{R}{r}\right)}^{l}{\bar{P}}_{lm}(\sin \phi )[{\bar{C}}_{lm}\cos (m\lambda )+{\bar{S}}_{lm}\sin (m\lambda )],$$

where GM is the mass parameter of the Moon, *l* is the spherical harmonic degree, *m* is the order, ${\bar{P}}_{{lm}}$ are the normalized associated Legendre polynomials, ${\bar{C}}_{{lm}}$ and ${\bar{S}}_{{lm}}$ are the normalized spherical harmonic coefficients, *R* is the reference radius of Moon (1,738 km), *λ* is longitude, *ϕ* is latitude and *r* is the radius evaluated at the spacecraft position relative to a moon’s body-fixed frame. In this formulation, zonal coefficients are defined as ${\bar{J}}_{l}=-{\bar{C}}_{l0}$. The gravity field is modelled in the lunar principal axis frame^1^. As we are assuming that the origin of the Moon’s body-fixed frame is defined to be the Moon’s COM, the degree-1 coefficients are identically zero. The unnormalized spherical harmonic coefficients, $({C}_{{lm}},{S}_{{lm}})$, are related to the normalized spherical harmonic coefficients as follows: $({\bar{C}}_{{lm}},{\bar{S}}_{{lm}})=({C}_{{lm}},{S}_{{lm}})/{N}_{{lm}}$, where the normalization factor ${N}_{{lm}}$ is defined as:

2 $${N}_{lm}=\sqrt{\frac{(l-m)!(2-{\delta }_{0m})(2l+1)}{(l+m)!}}.$$

The Moon’s tidal gravity field is modelled as corrections to the spherical harmonic coefficients^59–61^:

3 $$\Delta {C}_{lm}-{\rm{i}}\Delta {S}_{lm}=\frac{{k}_{lm}}{2l+1}\sum _{j}\frac{{{\rm{GM}}}_{j}}{{\rm{GM}}}\frac{{R}^{l+1}}{{r}_{j}^{l+1}}{P}_{lm}(\sin {\phi }_{j}){{\rm{e}}}^{-{\rm{i}}m{\lambda }_{j}},$$

where GM_*j*_ is the mass parameter of body *j* and $({\lambda }_{j},{\phi }_{j},{r}_{j})$ represents the longitude, latitude and distance of the body *j* in the lunar body-fixed frame^5,17^. Equation (3) accounts for Earth–Moon tides, where *j* = 1 arises from both the Moon’s orbital eccentricity and its fixed roughly 6.7° obliquity around the Earth, as well as *j* = 2 for the Sun–Moon tides. The Sun–Moon tides generate forcing potentials that are roughly 5–10 times and 1,000 times smaller than the Earth–Moon tides at *l* = 2 and *l* = 3, respectively, and act over a shorter period. Moreover, for a laterally heterogeneous Moon, combinations of Sun–Moon and Earth–Moon tides induce temporal changes in the *k*_3*m*_ based on equation (3). However, these variations are 2–3 times smaller than the effective uncertainties in *k*_3*m*_ derived from gravity field inversions (Table 1) and are, therefore, disregarded in our analysis.

The gravity field of the Moon is usually determined by means of a least-squares estimation technique^62,63^. Several lunar gravity models have been delivered previously using least-squares estimation, with varying resolutions^5–8,22–24^. Achieving a high-resolution gravity field is important for improving the spatial resolution, as well as for improving the long-wavelength spectrums of the gravity, in particular *l* = 2 and *l* = 3, which are important for computing the lunar moments of inertia^10^ and gravitational tidal Love numbers *k*_2*m*_ and *k*_3*m*_ (refs. ^5,7^). Considering the intrinsic data quality and the ground track coverage, we recover a degree-1,800 field called GL1800F, which takes out almost all of the available gravity signatures with the full wavelength surface resolution of 6 km (that is, roughly 2π*R*/*l*).

The gravity field estimation is typically a two-step process^64–67^. First, the trajectory of each arc is computed through an orbit determination process by estimating the arc-dependent parameters, such as spacecraft initial states and non-gravitational periodic accelerations to account for spacecraft thermal radiation forces. Specifically, estimated arc-dependent (that is, local) parameters are the spacecraft state (unconstrained), three solar pressure scale factors (along the spacecraft–Sun direction at 1 and 0.1% for the two other normal directions) and 15 periodic acceleration coefficients per spacecraft in radial, transverse and normal directions every orbit (roughly 2 h). The a priori uncertainties of the periodic accelerations (constant, once-per-rev, twice-per-rev) depend on the spacecraft orbit geometry relative to the Sun (fig. 17 of ref. ^57^). The maximum initial uncertainties are at 1 × 10^−12^ km s^−^^2^ for orbits with the most lunar shadowing and a minimum of 3 × 10^−14^ km s^−^^2^ for the spacecraft always in full Sun. Also, during full Sun, radial components are removed, as well as twice-per-rev for the other two directions. The weighting scheme used for the GL1800F solution was identical to the weight scheme used for the GL0900D solution^6^. For the PM arcs, the 5-s KBRR data were weighted at 30 nm s^−1^, except for 19-MAY-2012 and 22-MAY-2012 arcs that were weighted at 60 nm s^−1^ due to signal multipaths off the lunar surface. For the XM arcs, the 2-s KBRR data were weighted at 50 nm s^−1^. The two-way DSN S-band Doppler data were weighted at 0.1 mm s^−1^. These weight values provide for correct scaling of the gravity field uncertainties for *n* > 100 based on differences of gravity solutions (for example, GL1800F and GL1500E). The second step involves solving for the global parameters, such as the gravity field, that are common to all arcs. For GL1800F, the estimated global parameters were Moon’s degree-1,800 gravity field, gravitational tidal Love numbers *k*_2*m*_ and *k*_3*m*_, and the amplitudes of monthly periodic lunar core parameters. More details of least-squares estimation technique and filter setup are presented in the simulation results^57^ and the PM results^5,7^.

Computing a degree-1,800 field involves solving for roughly 3.24 million parameters. This is an extremely intensive numerical problem and requires careful implementation of generating partial derivatives, packing square-root information filter arrays, and inverting the resulting 39-TB upper-triangular square-root information filter matrix. The computation of GL1800F was done on NASA Ames Pleiades Supercomputers using 700 Haswell nodes, which has 20 cores with 128 GB of memory per node.

**Extended Data Fig. 6:**
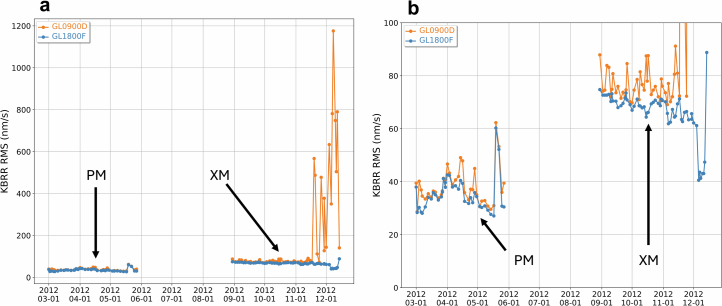
Post-fit residuals of GRAIL KBRR data using GL0900D and GL1800F. **a**, Post-fit residuals of Primary Mission’s 5-second KBRR data using GL1800F have an average RMS of approximately 35 nm/s. Post-fit residuals of Extended Mission’s 2-second KBRR data using GL1800F have an average RMS of approximately 65 nm/s. The GL1800F solution fits the KBRR data much better than GL0900 for the PM and XM phases, by as much as a factor of 28 for the 08-DEC-2012 arc. **b**, Same as **a**, but zoomed in.

The resulting GL1800F almost completely flattens the postfit residuals of all the KBRR and DSN data, indicating that almost all gravity signatures are taken out from the available GRAIL data. The root-mean-square (r.m.s.) values of the KBRR residuals are shown in Extended Data Fig. 6. The postfit residuals of PM’s 5-s KBRR data have an average r.m.s. of about 35 nm s^−1^, with a minimum of 27 nm s^−1^ for the 17-MAY-2012 arc and the postfit residuals of XM’s 2-s KBRR data have an average r.m.s. of about 65 nm s^−1^. The overall noise characteristic of the KBRR data is mostly dominated by the Lunar Gravity Ranging System’s thermal noise. Compared to GL900D (ref. ^6^), the postfit residuals have improved everywhere, including a factor of 28 improvement for the 08-DEC-2012 arc in the last month of the XM. A few arcs during the last 2 weeks of the XM show the postfit residuals of about 40 nm s^−1^, which is much less than postfit residuals of the earlier phase of the XM. This is because the separation distance was much less for these periods, yielding a much higher signal-to-ratio for the KBRR data. The postfit residuals of both PM and XM’s 10-s DSN S-band data have an average of roughly 0.1 mm s^−1^, which is good compared to other planetary orbiters, due to the close Earth-to-Moon distance and the high-accuracy Earth media calibrations available from the DSN.

Processing GRAIL data requires a background model for the Moon’s orbit and orientation. We used the JPL’s Development Ephemeris (DE) series, which provides the time series of the ephemeris and orientation of the Moon. In this study, we considered both DE430 (ref. ^21^) and DE440 (ref. ^1^), which are based on the GRAIL-derived lunar gravity field. DE440 includes seven more years of lunar laser ranging data and an improved lunar gravity model^1^.

The gravity field coefficients for a degree-1,800 solution require a constraint of some type; without one, the coefficients become unrealistically large. Previous solutions, for example, used a power law constraint for part of the spectrum with *l* > 600 (GL0900D)^6^ or a topographic rank minus one (RM1) constraint^23^. We first computed an unconstrained degree-1,800 solution based on DE430, and another degree-1,800 solution was then computed by applying a different topographic constraint for *l* > 600 (also based on DE430). In this constraint, the a priori values of the spherical harmonic gravity coefficients are given by the gravity field coefficients determined from topography expanded to the 23rd power (ref. ^68^) with increasing density with depth. This is accomplished by first computing a constant density gravity from topography $[{\rm{GT}}({\rho }_{0})]$ and then secondly by scaling the coefficients of each harmonic degree to the effective density for that given degree $({{\rm{GT}}}_{l}=({\rho }_{l}/{\rho }_{0}){\rm{GT}}({\rho }_{0}))$. The density versus degree was determined with a power law fit of the effective density from degrees 200 to 600 and then extrapolated to a density of 2,200 kg m^−^^3^ at degree 1,800 (Extended Data Fig. 3a,b, orange line). The a priori uncertainty of the constrained gravity harmonic coefficients were assumed to be 30% of the a priori values. The constraint is applied to smooth the coefficients over the areas where the solution is not as well determined. Using this constrained degree-1,800 gravity field as the background model, the GL1800F was computed by estimating degree-1200 spherical harmonic coefficients using DE440. This partial solution update was done to use the latest-available lunar orbit and orientation model and to avoid recomputation of a full degree-1,800 solution. The GL1800F solution is JPL’s first public release of a degree-1,800 lunar gravity field and is the highest resolution lunar gravity field published thus far (full wavelength surface resolution of 6 km).

The GL1800F gravity field is tied to the DE440 lunar orientation and is derived in the lunar principal axis frame. To be consistent with lunar cartographic products, GL1800F is also available in the mean-Earth frame. As the rotation from the principal axis-to-mean-Earth frame is defined as a fixed rotation^1^, the most straightforward way of computing the lunar gravity field in the mean-Earth frame is simply rotating the PA-based gravity field to the mean-Earth frame. This was accomplished with the available SHTOOLS software^69^. The 3–2–3 Euler rotation angles are:

(179.766217602292°, 0.021840987056123°, −179.785033451244°) for DE430,

(179.778382756033°, 0.0218596924458581°, −179.797230715235°) for DE440.

Both the GL1800F gravity field in the principal axis and mean-Earth frames are available through NASA’s Planetary Data System, and they are equivalent at the numerical noise level. For the Moon, the mean-Earth system is recognized as the international standard for lunar surface coordinates, as recommended by the International Astronomical Union’s Working Group on Cartographic Coordinates and Rotational Elements^70^. For tracking features on the lunar surface using cartographic products, such as surface mapping and optical or terrain-relative navigation, using the mean-Earth frame is therefore strongly recommended^71^.

Extended Data Fig. 3c,d show the r.m.s. gravity spectrum and errors of the constrained and unconstrained GL1800F solutions. The gravity spectrum shows a significant improvement of GL1800F (green) over GL0900D (blue) and GL1500E (orange). The constraint of the higher degrees of the lunar gravity field to within 30% of their expected values on the basis of topography gives similar results to previous RM1 solutions, although with a tighter *λ* = 10 constraint (fig. 9 in ref. ^23^). The Bouguer spectrum of GL1800F (cyan) crosses its error spectrum (green) at about *l* = 850, indicating that the solution is on average accurate to about *l* = 850. The independent solution GRGM1200B with *λ* = 1.0 (ref. ^23^) is also shown for comparison (yellow). Extended Data Fig. 3e,f show the correlations of gravity with gravity from topography for GL0900D, GL1500E, GRGM1200B and GL1800F, as well as the unconstrained GL1800F solution for comparison. Similar to the gravity spectrum, the correlation of GL1800F is significantly higher for *l* > 600 over GL0900D and GL1500E because of the topographic constraint.

**Extended Data Fig. 7:**
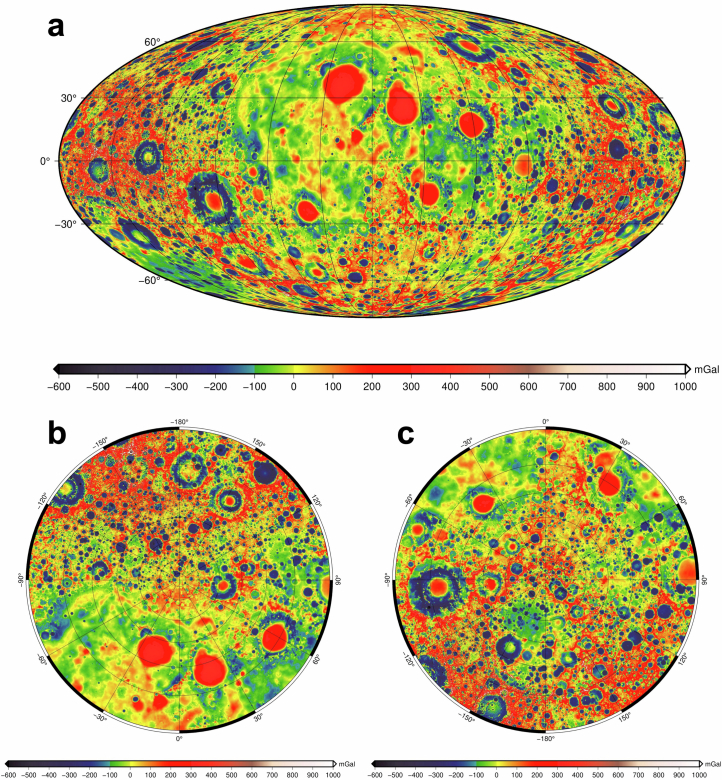
Global surface radial acceleration of the GL1800F lunar gravity field using spherical harmonics *l* = 2-600 and excluding ${\bar{{\boldsymbol{J}}}}_{{\bf{2}}}$ (positive downward). **a**, Mollweide projection (max = 1676 mGals, min = −930 mGals). **b**, Stereo projection of the northern hemisphere. **c**, Stereo projection of the southern hemisphere.

**Extended Data Fig. 8:**
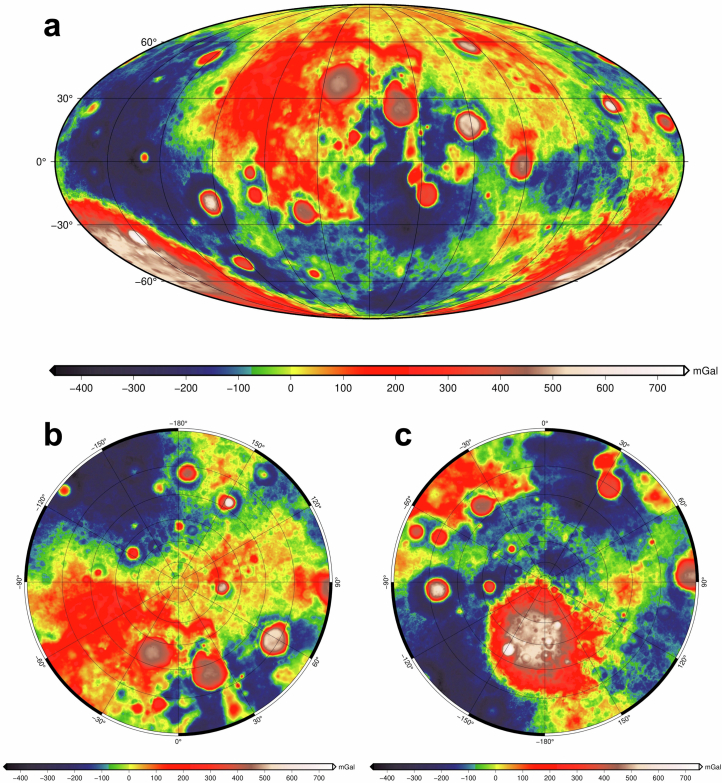
Bouguer anomaly map of GL1800F lunar gravity field using spherical harmonics *l* = 2-600 (positive downward). **a**, Mollweide projection (max = 715 mGals, min = −440 mGals). **b**, Stereo projection of the northern hemisphere. **c**, Stereo projection of the southern hemisphere.

Extended Data Fig. 7 shows the surface radial acceleration (positive downwards) of the lunar gravity field GL1800F solution projected onto the reference sphere of 1,738 km for the harmonic coefficients truncated at degree 600, excluding ${\overline{J}}_{2}$ (maximum, 1,676 milliGal (mGal, unit of acceleration); minimum, −930 mGal). Extended Data Fig. 7a shows the Mollweide projection, Extended Data Fig. 7b shows the stereo projection of the northern hemisphere for 90° to 0° latitude and Extended Data Fig. 7c shows the stereo projection of the southern hemisphere for −90° to 0° latitude. Extended Data Fig. 8 shows the Bouguer gravity anomaly map of the lunar gravity field GL1800F solution projected onto the reference sphere of 1,738 km for the harmonic coefficients truncated at degree 600 (maximum 715 mGal, minimum −440 mGal). The Bouguer map was computed by differencing the surface radial acceleration of GL1800F and the gravity from topography, which was computed using the density of 2,372 kg m^−^^3^ for harmonic coefficients up to *l* = 600, whereas the extrapolated effective density curve (orange) was used for coefficients *l* > 600. Extended Data Fig. 8a shows the Mollweide projection, Extended Data Fig. 8b shows the stereo projection of the northern hemisphere for 90° to 0° latitude and Extended Data Fig. 8c shows the stereo projection of the southern hemisphere for −90° to 0° latitude.

**Extended Data Fig. 9:**
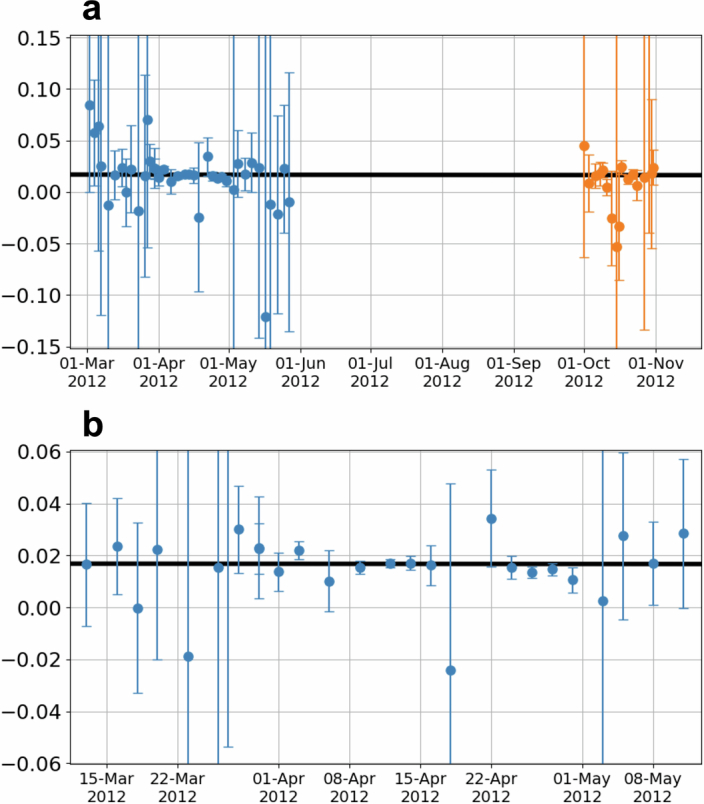
Estimated *k*_3_ per arc (e.g., 2-3 days long) to show the variability in the solution. **a**, The blue points show the *k*_3_ solution from all PM arcs and orange points show the *k*_3_ solution from October XM arcs. During April and October, the GRAIL spacecraft did not go through the shadow, minimizing the non-gravitational effects. The solid black line shows the nominal value *k*_3_ = 0.0163. **b**, Same as **a**, but zoomed in for April arcs, showing consistently high estimates of *k*_3_ for each arc.

The nominal GL1800F includes *k*_2*m*_ and *k*_3_ and their recovered values are shown in Table 1, showing when *k*_3_ = *k*_3*m*_, the recovered *k*_3_ = 0.0163 ± 0.0007 (that is, case 1). Compared to the *k*_3_ value expected for a spherically symmetric Moon (that is, *k*_3_ = 0.00945)^10,27^, our estimated *k*_3_ is about 72% larger. Extended Data Fig. 9 shows the variability of *k*_3_ per arc, where each arc is 2–3 days long. In these solutions, all the local parameters (for example, spacecraft state, solar pressure scale factors, non-gravitational period accelerations) are estimated together with *k*_3_ for each arc assuming the same data weights as mentioned above and with GL1800F as the nominal gravity field. The blue points show the *k*_3_ solutions for all PM arcs. During April and October, the GRAIL spacecraft were not occulted by the Moon when observed from the Sun, thereby minimizing non-gravitational forces, as indicated by the a priori constraint values mentioned above. Thus, during this period, the large *k*_3_ value is clearly recovered. The data from September, November and December are not shown because of the strong non-gravitational effects due to low spacecraft altitude and going through shadows. Table 1 also shows individual *k*_3*m*_ when values at each order are estimated independently (that is, case 2). We have analysed the sensitivity to *k*_4*m*_, but there was no significant sensitivity in the GRAIL dataset for degrees higher than *l* = 3.

### Inversion procedure

To constrain the structure of the lunar interior based on GL1800F, we carry out a Bayesian inversion using MCMC with a Metropolis–Hastings sampling algorithm using PyMC. For our inversion, we vary elastic parameters relative to a reference lunar interior to fit observed *k*_2m_ and *k*_3m_ Love numbers (Table 1)^72^. Our detailed procedure is described below.

Our reference model incorporates both 1D structure and lateral variations in crustal thickness (and density) a priori. We extract 1D (that is, radial) elastic parameters and density for reference models from ref. ^27^ that constrains lunar structure using the satellite’s mean density and seismic wave arrival times (see Extended Data Table 1 for assumed mean shear modulus, bulk modulus and density values for each internal layer). Whereas moment of inertia can be used to further refine the interior structure of reference models, this constraint primarily informs the size of the lunar core (at more than 1,400 km depth) and therefore has minimal impact on interpretations of *k*_2*m*_ or *k*_3*m*_ in this study^29,10,73,74^ (Fig. 1b). Nominal viscosity for each layer is set to effectively infinite values (10^30^ Pa s). To account for variations in crustal structure^25^, we vary spherical harmonic coefficients describing shear modulus, bulk modulus and density for two adjacent internal layers (extending from 0 to 34 and 34 to 62 km in depth) following the method described in ref. ^20^ (see Extended Data Fig. 4a for assumed coefficient values).

We consider both 1D and 3D perturbations to the shear modulus and do not consider changes in density (that is, which may violate constraints from static gravity measurements, Fig. 3a or ref. ^25^) or bulk modulus (which have a negligible effect on ${k}_{2m}$ or ${k}_{3m}$, see ref. ^20^). As shear modulus (*μ*) is related to shear wave speed (*V*_s_) and density (*ρ*) through $\mu =\rho {V}_{{\rm{s}}}^{2}$, our approach effectively perturbs *V*_s_ while maintaining fixed *ρ* within each model layer. We consider a total of 34 parameters: 30 parameters describing 3D variations of shear modulus $({\mu }_{{lm}}^{{\prime} })$ (that is, *l* = 1–3 spherical harmonic coefficients for the crust and mantle) and four parameters describing 1D structure $({\mu }_{00}^{{\prime} })$ (that is, *l* = 0 coefficients for the crust, the 34- to 734-km region, the 734- to 1,257-km region and the 1,257- to 1,407-km region). These model parameters are sampled as coefficients for spherical harmonic basis functions that comprise the base-10 logarithm (denoted by the symbol ′) of the ratio of the spatially variable shear modulus *μ* to that of the reference model *μ*_ref_ for each internal layer:

4 $${\log }_{10}\frac{\mu }{{\mu }_{{\rm{ref}}}}=\mathop{\sum }\limits_{l=0}^{3}\mathop{\sum }\limits_{m=-l}^{l}{\mu }_{lm}^{{\prime} }{Y}_{lm}(\theta ,\lambda ),$$

where ${\mu }_{{lm}}^{{\prime} }$ are sampled coefficients and ${Y}_{{lm}}(\lambda ,\theta )$ are real form, ortho-normalized spherical harmonic basis functions (*λ* is the longitude, and *θ* is the colatitude in the COM reference frame):

5 $${Y}_{lm}(\theta ,\lambda )=\left\{\begin{array}{c}\sqrt{\frac{1}{2{\rm{\pi }}}}{N}_{lm}{\bar{P}}_{lm}(\cos \theta )\cos (m\lambda ),{\rm{for}}\,m > 0,\\ \sqrt{\frac{1}{4{\rm{\pi }}}}{N}_{lm}{\bar{P}}_{lm}(\cos \theta ),{\rm{for}}\,m=0,\\ \sqrt{\frac{1}{2{\rm{\pi }}}}{N}_{l| m| }{\bar{P}}_{lm}(\cos \theta )\sin (| m| \lambda ),{\rm{for}}\,m < 0.\end{array}\right.$$

We expand shear modulus structures for accepted candidate models into spherical harmonics (in postprocessing) to compute percentage perturbations *μ*_*lm*_ (that is, values presented in Fig. 2 and Extended Data Table 2):

6 $$\frac{\mu }{{\mu }_{{\rm{ref}}}}=1+{10}^{-2}\mathop{\sum }\limits_{l=0}^{3}\mathop{\sum }\limits_{m=-l}^{l}{\mu }_{lm}{Y}_{lm}.$$

The Maxwell viscosity *η* of the 1,257- to 1,407-km depth layer is computed by assuming *l* = 0 perturbations to the shear modulus of this layer correspond to changes in this layer’s effective shear modulus *μ*_eff_ (that is, the amplitude of the complex shear modulus at the monthly timescale):

7 $$\eta =\frac{{\mu }_{{\rm{ref}}}}{\omega \sqrt{\left(\frac{{\mu }_{{\rm{ref}}}}{{\mu }_{{\rm{eff}}}}-1\right)}}$$

where *ω* = 2.661 × 10^−6^ s^−1^ is the angular frequency corresponding to the lunar sidereal monthly period and

8 $${\mu }_{{\rm{eff}}}={\mu }_{{\rm{ref}}}(1+{10}^{-2}\sqrt{4{\rm{\pi }}}{\mu }_{00}).$$

Coefficients for sampled *l* = 1 to *l* = 3 structure (that is, ${\mu }_{{lm}}^{{\prime} }$ in equation (4)) are assumed to have uniform (that is, flat) prior probability distributions. Note that our method does not necessarily require the use of spherical harmonics as basis functions for calculations. For example, a 2–3% amplitude spherical cap (placed at the sub-Earth point) spanning the nearside hemisphere is sufficient to explain the observed 2–3% (*l* = 1, *m* = 1) variation in mantle shear modulus derived in this work. By contrast, we assume sampled ${\mu }_{00}^{{\prime} }$ coefficients have Gaussian prior distributions with variances extracted from ref. ^27^. We separately sample ${\mu }_{00}^{{\prime} }$ coefficients for regions between 34- to 734 km-, 734- to 1,257-km and 1257- to 1,407-km depths (that is, the lunar low-velocity zone or LVZ) to account for differences in mean shear modulus uncertainty for these regions (the variance for ${\mu }_{00}^{{\prime} }$ in the LVZ is set to infinity)^27^. Increasing the variances of the ${\mu }_{00}^{{\prime} }$ coefficients tends to distribute reductions in the mean shear modulus (required to explain high degree-2 Love numbers, Fig. 1a) to each layer, thereby increasing the inferred Maxwell viscosity of the LVZ from equation (7). Moreover, changing the assumed model for viscoelasticity (for example, from Maxwell to Kevin–Voigt) alters the inferred viscosity value(s) associated with a roughly 97% reduction in the effective shear modulus of the LVZ at the sidereal monthly period (relative to the effective shear modulus at seismic timescales, Extended Data Table 2) by up to an order of magnitude. Note that all sampled coefficients ${\mu }_{{lm}}^{{\prime} }$ (that is, not just ${\mu }_{00}^{{\prime} }$) could, in principle, represent changes in effective shear modulus *μ*_eff_ and (by extension) variations in internal viscosity. However, only significant lateral changes in viscosity in the LVZ (that is, due to variations in temperature near the lunar mantle’s solidus in this region) are likely to drive substantial variation in *μ*_eff_ as per equation (7). This suggests that the inferred 2–3% variation in mantle shear modulus may reflect a pronounced lateral viscosity variation within the LVZ. However, without extra constraints, it remains unclear to what extent inferred asymmetries localize to any region of the deep interior or are indicative of a broader (for example, mantle wide) anomaly (main text discussion).

We generate an ensemble of internal structure models for Markov chains by sampling prior probability distributions for model parameters and forward computing Love numbers using these values. Each ensemble consists of roughly 1,000 individual accepted model realizations (that is, 50,000 samples total from 50 walkers). To speed up convergence, we consider only shear modulus perturbations and sample harmonic coefficients describing this structure up to *l* = 3 for inversions (earlier discussion). We also adopt an adaptive sampling approach (the ‘tune’ functionality in PyMC) that dynamically adjusts step sizes on the basis of the sensitivity of model outputs to input parameters^72^. We visually inspect Markov chains to discard initial burn-in steps (that is, typically the first roughly 10–20% of samples) and terminate inversions when parameter autocorrelations are greater than 0.99. Walker positions are updated on the basis of a likelihood function that considers only degree-2 and degree-3 Love number values (case 2 of Table 1):

9 $$\text{log}L\propto -\frac{1}{2}{({\bf{X}}-{\bf{Y}})}^{T}{\Sigma }^{-1}({\bf{X}}-{\bf{Y}}),$$

where **X** is the vector of observed Love numbers and **Y** is the vector of model-predicted Love numbers. Σ is a matrix that considers both observational and model covariances $(\Sigma ={\Sigma }_{{\rm{obs}}}+{\Sigma }_{{\rm{mod}}})$. Both Σ_obs_ and Σ_mod_ are assumed to be diagonal (that is, each Love number observation and model parameter is independent). Note that differences between observations and modelled Love numbers (**X** − **Y**) yield maximum *L* values of roughly 0.8 (out of a possible 1) across our ensemble of accepted models, supporting our interpretation that *k*_3*m*_ observations can be adequately explained by a nearside–farside asymmetry in the interior (Extended Data Fig. 2). Other system constraints (for example, mean density, moment of inertia or quality factors) are not incorporated into vectors **X** or **Y** in equation (9).

### Modelling tidal deformation

We compute lunar Love numbers using the semi-analytic spectral method LOV3D (ref. ^3^), which solves mass conservation, momentum and Poisson’s equations in the Fourier domain for a laterally heterogeneous body subject to tidal loading:

10 $${\rho }^{{\prime} }=-{\rho }_{0}(\nabla \cdot {\bf{u}})-{\bf{u}}\cdot \nabla {\rho }_{0}$$

11 $$\nabla \cdot {\sigma }^{{\prime} }-{\rho }_{0}\nabla (g{\bf{u}}\cdot {{\bf{e}}}_{r})+g{\rho }_{0}(\nabla \cdot {\bf{u}}){{\bf{e}}}_{r}-{\rho }_{0}\nabla {\varphi }^{{\prime} }=0$$

12 $${\nabla }^{2}{\varphi }^{{\prime} }=4\pi G{\rho }^{{\prime} }$$

where **u** is the displacement vector, *σ*′ is the incremental material stress tensor, **e**_r_ is the radial unit vector, *g* the gravitational acceleration of the unperturbed body, ${\rho }^{{\prime} }$ is the incremental local density, *G* is the universal gravitational constant, *ρ*_0_ is the density of the unperturbed body and ${\varphi }^{{\prime} }$ is gravitational potential arising from tides and mass movement driven by deformation. We use the constitutive equation for isotropic linear elasticity to relate *σ*′ and **u**:

13 $$\sigma {\prime} =\left(\kappa -\frac{2}{3}\mu \right)(\nabla \cdot {\bf{u}})I+\mu (\nabla {\bf{u}}\cdot \nabla {{\bf{u}}}^{T})$$

where *I* is the identity matrix and *μ* and *κ* are the shear and bulk moduli. We find minimal differences (less than 0.01%) between results produced by our methodology and numerical (that is, finite-element) solutions for displacement on a laterally heterogeneous moon subject to tidal loading. Moreover, our results for perturbations to the lunar gravity field for lunar interiors with *l* = 1, *m* = 1 shear modulus structure are broadly consistent with results presented in fig. 1 of ref. ^4^.

We discount the influence of polar motion—the movement of a planetary body’s rotational axis relative to its surface—on calculations of degree-2 and degree-3 Love numbers. This simplification is based on our expectation that the body tides considered in our work induce only minimal changes to the Moon’s moment of inertia tensor over the GRAIL observation period. To verify this assumption, we computed the amplitude of polar motion resulting from a static degree-2, order-1 bulge of 1 cm height. The resulting value, roughly 10^−3^ degrees, is orders of magnitude smaller than the Moon’s roughly 6.7° obliquity (that is, which is the dominant driver of degree-2, order-1 forcing for the Moon). Nonetheless, we expect that polar motion may substantially influence longer-term response to surface loading (for example, fig. 3a,b in ref. ^75^).

LOV3D explicitly computes coefficients ${K}_{{l}^{{\prime} },{m}^{{\prime} }}^{l,m}$, or ‘Extended Love numbers’ (distinct from the ‘traditional’ Love numbers *k*_*lm*_ described in equation (3)). ${K}_{{l}^{{\prime} },{m}^{{\prime} }}^{l,m}$ represents coupling between forcing at one harmonic (at *l*′, *m*′) and gravitational response to this forcing at another harmonic (at *l*, *m*) for a given interior structure. For a laterally heterogeneous body, equation (3) can be derived considering a general expression for perturbations to gravity field coefficients $\Delta {C}_{{lm}}$ and $\Delta {S}_{{lm}}$ in terms of real form ${K}_{{l}^{{\prime} },{m}^{{\prime} }}^{l,m}$:

14 $$\begin{array}{c}\Delta {C}_{lm}-{\rm{i}}\Delta {S}_{lm}=\mathop{\sum }\limits_{{l}^{{\prime} }=2}^{3}\mathop{\sum }\limits_{{m}^{{\prime} }=0}^{{l}^{{\prime} }}\frac{1}{2{l}^{{\prime} }+1}\sum _{j}\frac{{{\rm{G}}{\rm{M}}}_{j}}{{\rm{G}}{\rm{M}}}\frac{{R}^{{l}^{{\prime} }+1}}{{r}_{j}^{{l}^{{\prime} }+1}}{P}_{{l}^{{\prime} }{m}^{{\prime} }}(\sin {\phi }_{j})\\ \,\,\,\,\,\,\,[({K}_{{l}^{{\prime} },{m}^{{\prime} }}^{l,m}\cos ({m}^{{\prime} }{\lambda }_{{\rm{j}}})+{K}_{{l}^{{\prime} },-{m}^{{\prime} }}^{l,m}\sin ({m}^{{\prime} }{\lambda }_{{\rm{j}}}))\\ \,\,\,\,\,\,\,-{\rm{i}}({K}_{{l}^{{\prime} },{m}^{{\prime} }}^{l,-m}\cos ({m}^{{\prime} }{\lambda }_{{\rm{j}}})+{K}_{{l}^{{\prime} },-{m}^{{\prime} }}^{l,-m}\sin ({m}^{{\prime} }{\lambda }_{{\rm{j}}}))].\end{array}$$

In this work, we make the following simplifications:

1. Gravity field inversions assume that perturbations $\Delta {C}_{30}$, ${\Delta C}_{31}$, $\Delta {C}_{32}$, $\Delta {C}_{33}$, ${\Delta S}_{31}$, $\Delta {S}_{32}$ and $\Delta {S}_{33}$ are not affected by coupling that is temporally out of phase with forcing at these harmonics. We correspondingly set ${K}_{{l}^{{\prime} },{m}^{{\prime} }}^{l,-m}$, ${K}_{{l}^{{\prime} },-{m}^{{\prime} }}^{l,m}$ and ${K}_{\mathrm{2,1}}^{\mathrm{3,1}}$, ${K}_{\mathrm{2,1}}^{\mathrm{3,3}}$, ${K}_{\mathrm{2,0}}^{\mathrm{3,0}}$, ${K}_{\mathrm{2,0}}^{\mathrm{3,2}}$, ${K}_{\mathrm{2,2}}^{\mathrm{3,0}}$, ${K}_{\mathrm{2,2}}^{\mathrm{3,2}}$, ${K}_{2,-2}^{3,-2}$, ${K}_{2,-1}^{3,-1}$ and ${K}_{2,-1}^{3,-3}$ to zero a priori.
2. To improve computational efficiency, we assume ${K}_{{l}^{{\prime} },{m}^{{\prime} }}^{l,m}={K}_{{l}^{{\prime} },-{m}^{{\prime} }}^{l,-m}$. However, we note that limited MCMC results (roughly 5,000 accepted candidate models) indicate that separate computations of Love numbers that assume ${K}_{{l}^{{\prime} },-{m}^{{\prime} }}^{l,-m}\ne {K}_{{l}^{{\prime} },{m}^{{\prime} }}^{l,m}$ does not substantially (less than 1%) alter results presented in Figs. 2–4 and Extended Data Figs. 4b and 5.

On the basis of these assumptions, we can rewrite equation (14):

15 $$\Delta {C}_{lm}-{\rm{i}}\Delta {S}_{lm}=\mathop{\sum }\limits_{{l}^{{\prime} }=2}^{3}\mathop{\sum }\limits_{{m}^{{\prime} }=0}^{{l}^{{\prime} }}\frac{{K}_{{l}^{{\prime} },{m}^{{\prime} }}^{l,m}}{2{l}^{{\prime} }+1}\sum _{j}\frac{{{\rm{GM}}}_{j}}{{\rm{GM}}}\frac{{R}^{{l}^{{\prime} }+1}}{{r}_{j}^{{l}^{{\prime} }+1}}{P}_{{l}^{{\prime} }{m}^{{\prime} }}(\sin {\phi }_{j}){{\rm{e}}}^{-{\rm{i}}{m}^{{\prime} }{\lambda }_{j}}.$$

Note that we can compute individual components of the tidal forcing potential ${V}_{{l}^{{\prime} }{m}^{{\prime} }}$ from equation (15):

16 $${V}_{{l}^{{\prime} }{m}^{{\prime} }}=\frac{1}{2{l}^{{\prime} }+1}\sum _{j}\frac{{{\rm{GM}}}_{j}}{{\rm{GM}}}\frac{{R}^{{l}^{{\prime} }+1}}{{r}_{j}^{{l}^{{\prime} }+1}}{P}_{{l}^{{\prime} }{m}^{{\prime} }}(\sin {\phi }_{j}){{\rm{e}}}^{-{{\rm{i}}m}^{{\prime} }{\lambda }_{j}}.$$

Comparing equations (16), (15) and (3), it becomes apparent that traditional Love numbers *k*_*lm*_ represent the ratio of tidal (that is, forcing) potentials at (*l*′, *m*′) and response at (*l*, *m*) (that is, ${V}_{{l}^{{\prime} }{m}^{{\prime} }}$ and ${V}_{{lm}}$ from equation (15)) scaled by ${K}_{{l}^{{\prime} },{m}^{{\prime} }}^{l,m}$:

17 $${k}_{lm}=\mathop{\sum }\limits_{{l}^{{\prime} }=2}^{3}\mathop{\sum }\limits_{{m}^{{\prime} }=0}^{{l}^{{\prime} }}{K}_{{l}^{{\prime} },{m}^{{\prime} }}^{l,m}\frac{{V}_{lm}}{{V}_{{l}^{{\prime} }{m}^{{\prime} }}}.$$

In the case of a spherically symmetric Moon, Extended Love numbers simplify to *k*_*lm*_ when $$l={l}^{{\prime} }$$ and $$m={m}^{{\prime} }$$ (for example, $${K}_{30}^{30}={K}_{31}^{31}\,=\,$$$${K}_{32}^{32}\,=\,{K}_{33}^{33}={k}_{3}$$ and $${K}_{20}^{20}={K}_{21}^{21}={K}_{22}^{22}={k}_{2}$$). However, for lunar interiors with degree-1 order-1 shear modulus variations, extra coupling terms (that is, $${K}_{20}^{31}$$, $${K}_{22}^{31}$$, $${K}_{22}^{33}$$, $${K}_{21}^{30}$$, $${K}_{21}^{32}$$) become significant such that $${k}_{31}\approx {K}_{31}^{31}+{K}_{20}^{31}{V}_{20}/{V}_{31}+{K}_{22}^{31}{V}_{22}/{V}_{31}$$ and $${k}_{33}\approx {K}_{33}^{33}+{K}_{22}^{33}{V}_{22}/{V}_{33}$$ (equation (17))^4^. Note that ref. ^4^. approximate $${V}_{3m}/{V}_{2m}=1/220$$. Using our exact numerical approach to compute tidal potentials (equation (16)) we find $${V}_{3m}/{V}_{2m}$$ ranges from roughly 1/200–1/300.

Using our MCMC method, we also examine whether an unconstrained spherically symmetric lunar interior (that is, with all 1-*σ* bounds on mean shear modulus values for internal layers in Extended Data Table 1 set to infinity) could theoretically explain observed Love number values. We find that these inversions require a 70–100% reduction in mean *μ*_eff_ within the uppermost 100–200 km of the Moon relative to values presented in Extended Data Table 1 to explain *k*_3*m*_ and *k*_2*m*_ in Table 1 (the required perturbations are shallow because *k*_3*m*_ are more sensitive to such perturbations than *k*_2*m*_). Such reductions suggest an unrealistically weak upper mantle or crust (for example, viscosities in equation (8) ranging from 10^9^ to 10^16^ Pa s, which falls at least five orders of magnitude below expected values for this region).

### Modelling temperature change

Our inference of a 100–200 K temperature difference (Fig. 3a) relies on linear relationships between temperature, shear modulus, density and composition (*β*, Δ*μ*/Δ*T*, Δ*ρ*/ΔFo–Fa, Δ*μ*/ΔFo–Fa) based on experimental studies of olivine. However, the lunar mantle probably contains at least roughly 5% pyroxene^76^, which may very slightly alter *β*, Δ*μ/*ΔT, Δ*ρ/*ΔFo–Fa and Δ*μ*/ΔFo–Fa (ref. ^77^). Phase changes could also cause deviations from this linear behaviour described by *β*, Δ*μ/*Δ*T*, Δ*ρ/*ΔFo–Fa and Δ*μ*/ΔFo–Fa; although such variations are probably confined to the LVZ. Minor phases (for example, ilmenite) may also be present in low concentrations throughout the lunar mantle^78^ but probably have a negligible effect on the results presented in Fig. 3a.

## Online content

Any methods, additional references, Nature Portfolio reporting summaries, source data, extended data, supplementary information, acknowledgements, peer review information; details of author contributions and competing interests; and statements of data and code availability are available at 10.1038/s41586-025-08949-5.

## Figures and Tables

**Extended Data Table 1:**
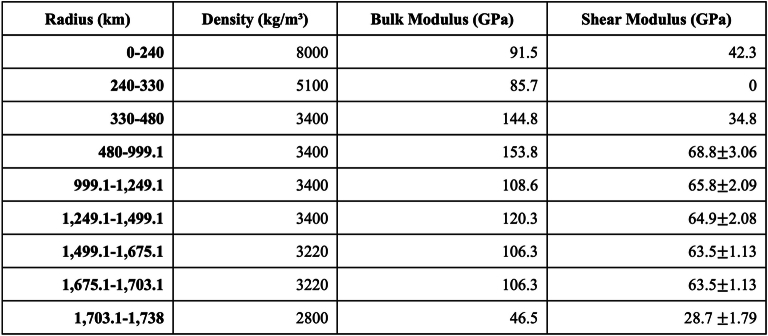
Radial (1D) elastic structure (density, bulk modulus, and shear modulus) assumed for reference models

Data extracted from ref. ^27^. Bounds for regions at radii >480 km denote 1-*σ* uncertainties from ref. ^27^.

**Extended Data Table 2:**
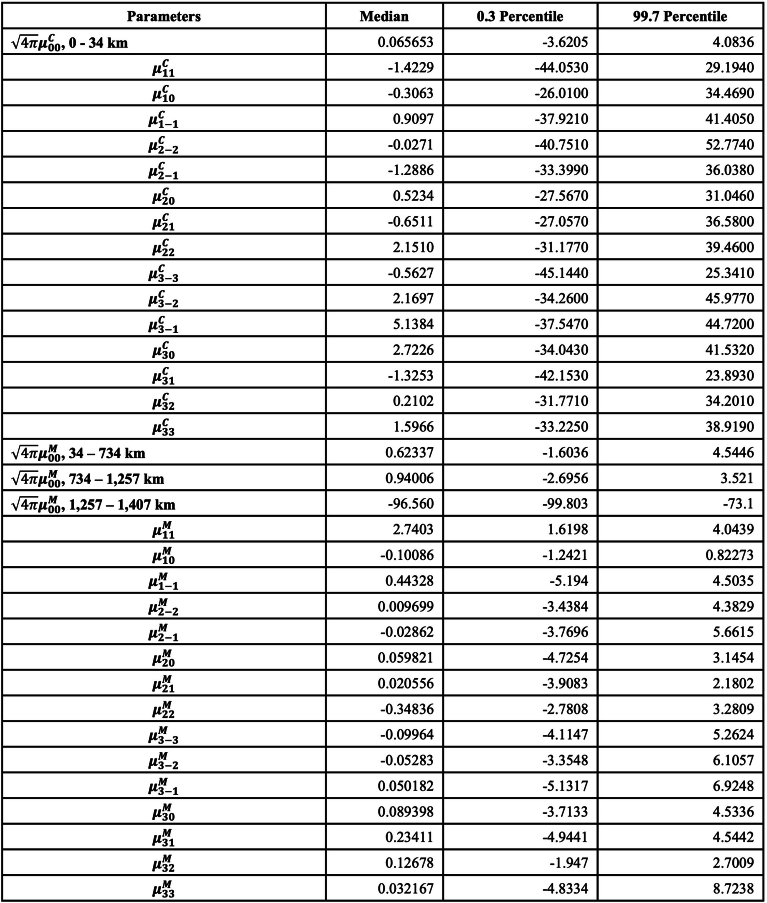
Inverted median, standard deviation, 0.3%, and 99.7% of coefficient values describing 3D structure in the lunar crust (superscript *C*, see Eqn. 7) and the lunar mantle (superscript *M*)

The $\sqrt{4\pi }$ factor is included to account for ortho-normalization of the degree-0 harmonic.

## Data Availability

The GRAIL data used to generate the results of this paper are available at the NASA Planetary Data System Geosciences Node (http://pds-geosciences.wustl.edu). The GL1800F gravity field tied to the principal axis frame and the mean-Earth frame can be downloaded from the GRAIL gravity archival directory (https://pds-geosciences.wustl.edu/grail/grail-l-lgrs-5-rdr-v1/grail_1001/shadr/).

## Code availability

The gravity recovery results presented in this study can be reproduced using the MONTE software available at NASA (https://montepy.jpl.nasa.gov). The LOV3D software is also available at GitHub (https://github.com/mroviranavarro/LOV3D_multi).
